# A Case with Repeated Recurrent Acute Coronary Syndrome due to Pseudoephedrine Use: Kounis Syndrome

**DOI:** 10.1155/2014/742905

**Published:** 2014-11-11

**Authors:** Metin Çeliker, Mustafa Tuncer, Ali Şekeralmaz

**Affiliations:** ^1^Ear, Nose and Throat Clinic, Rize Training and Research Hospital, Islampasa Mah., Sehitler Caddesi, No. 74, 53020 Rize, Turkey; ^2^Department of Cardiology, Faculty of Medicine, Yüzüncü Yıl University, 65080 Van, Turkey; ^3^Private Lokman Hekim Van Hospital, 65100 Van, Turkey

## Abstract

Allergic reaction-associated acute coronary syndrome picture is defined as Kounis syndrome. Although drug use is the most common cause of allergic reaction, foods and environmental factors may also play a role in the etiology. Herein, a case with acute coronary syndrome that developed two times at 8-month interval due to pseudoephedrine use for upper respiratory tract infection is presented.

## 1. Introduction

Kounis syndrome, which is an entity of allergic reaction-associated acute coronary syndrome, is defined as allergic angina/allergic myocardial infarction. This syndrome, which is related to the mediators released from mast cells, was first defined in 1991 by Kounis and Zavras [1]. In 1998, Braunwald [2] reported that vasospastic angina might be associated with allergic reactions. Etiological factors include drug use, foods, and environmental agents. Nonsteroid anti-inflammatory drugs, antibiotics, and sedative and anesthetic agents used for anesthesia are the drugs that have been most frequently associated with Kounis syndrome. To the best of our knowledge, the number of cases with acute coronary syndrome repeated due to the same agent is quite limited in the literature. Herein, a case with acute coronary syndrome that developed two times at 8-month interval due to pseudoephedrine use for upper respiratory tract infection is presented.

## 2. Case Presentation

**Figure 1:**
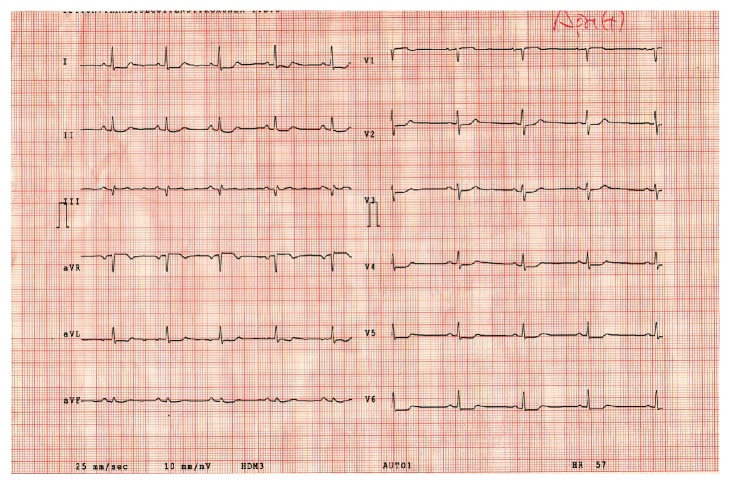
One mm ST depression in the leads D2, D3, aVF, V4, V5, and V612 on electrocardiography of the patient performed at the Emergency Service.

**Figure 2:**
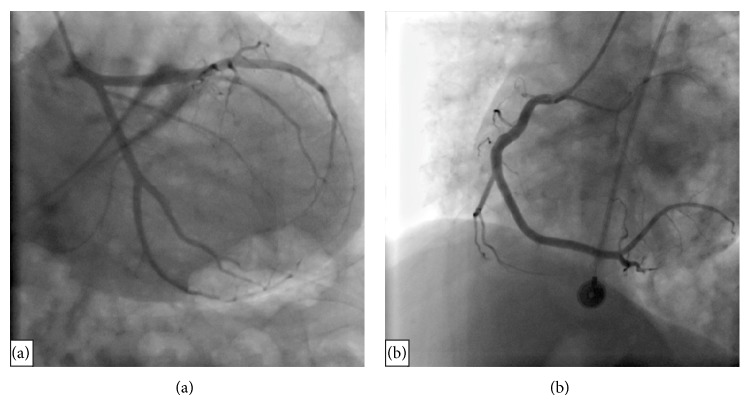
Normal appearance of coronary arteries: (a) left coronary artery system: left anterior descending artery and circumflex, (b) right coronary artery.

A 43-year-old male patient was admitted to our Emergency Service with chest pain and pain in the left arm lasting for 1 hour. On his physical examination, arterial blood pressure was 100/60 mmHg and pulse rate was 89/min. Cardiovascular auscultation revealed no additional heart sound or murmur. Respiratory system examination was unremarkable. Electrocardiography (ECG) of the patient that was performed in the Emergency Service revealed 90/min sinus rhythm and 1 mm ST depression on the leads D2, D3, aVF, V4, V5, and V6 (Figure 1). The patient was transferred to the Coronary Intensive Care Unit with the diagnosis of acute coronary syndrome without ST elevation. He received 300 mg acetylsalicylic acid (ASA) and 300 mg clopidogrel load via oral route and 5000 IU heparin via intravenous route, and nitroglycerin perfusion was started. Chest pain of the patient relieved and ST depression improved. Routine biochemistry analyses were performed. His chest pain restarted an hour later, and the patient was transferred to the catheterization laboratory for coronary angiography. He underwent right-left selective coronary angiography through the right femoral route. Coronary angiography revealed no significant stenosis in the left anterior descending artery and its branches, circumflex artery and its branches, and right coronary artery and its branches, which could explain the clinical picture (Figure 2). When his medical history was reevaluated, it was learned that he had received pseudoephedrine-containing drug for upper respiratory tract infection. On his blood biochemistry analyses, leukocyte count was 18.000/mm^3^, hematocrit was 44%, neutrophil was 86%, lymphocyte was 7.8%, eosinophil was 5.1%, troponin T level was 7.11 ng/mL (reference: 0.003–0.014 ng/mL), creatinine kinase (CK) level was 3,445 U/L, CK-MB was 515 U/L, postprandial blood glucose was 115 mg/dL, low-density lipoprotein level was 125 mg/dL, and creatinine level was 0.8 mg/dL. After coronary angiography, the patient who was transferred to the Intensive Care Unit for monitoring had no complaint under medical treatment. The patient, who had had upper respiratory system complaints 3 days ago and received a cold medicine containing pseudoephedrine, was considered to have Kounis syndrome since his coronary angiography was normal. Physical examination revealed no complaint or sign of allergic reaction. On his transthoracic echocardiographic examination, left ventricular ejection fraction was measured to be 65%. Regional wall motion impairment or any other pathology was not determined. Since his cardiac enzymes decreased and he had no complaints during monitoring, he was discharged on the 4th day with ASA 100 mg and atorvastatin 40 mg treatments; he was asked to return for a control visit one month later. The patient was readmitted to our Emergency Service eight months later with similar complaints and it was learned that he had again received a cold medicine containing pseudoephedrine. Anti-ischemic, antiaggregant, and anticoagulant treatments were commenced; ST-T segment changes on his ECG improved and his chest pain relieved.

Results of cardiac enzyme and other blood biochemistry analyses revealed that leukocyte count was 12,000/mm^3^, neutrophil was 44%, lymphocyte was 51%, eosinophil was 3.2%, troponin was 2.52 ng/mL (reference: 0.003–0.014 ng/mL), CK was 1,309 U/L, CK-MB was 145 U/L, and high-sensitivity C-reactive protein was 15 mg/L. As was at his previous admission, physical examination of the patients revealed no complaint or sign of allergic reaction. His transthoracic echocardiography demonstrated normal left ventricular function with no regional wall motion impairment.

Control coronary angiography was not performed since he was diagnosed with Kounis syndrome at his previous admission, his complaints clinically relieved, and he was hemodynamically stable. The patient, cardiac enzymes of whom decreased and who was free of cardiac complaints during monitoring, was discharged on the 3rd day with ASA and statin treatments. He was referred to the Immunology Polyclinic as he was considered to have allergic angina (Kounis syndrome).

## 3. Discussion

This paper presented a case, who developed Kounis syndrome (allergic angina/allergic myocardial infarction) for two times at 8-month interval each occurring after pseudoephedrine use for upper respiratory tract infection.

Etiological factors of Kounis syndrome include drugs, foods, and environmental agents (insect bite, snakebite, etc.). Although nonsteroidal anti-inflammatory drugs, antibiotics, sedative and anesthetic agents used for anesthesia, and iodine-contrast substances are the drugs that have been most frequently associated with Kounis syndrome [3–5], any kind of drug has the potential of inducing allergic reaction and causing Kounis syndrome. To the best of our knowledge, there is one case of pseudoephedrine-induced Kounis syndrome in the literature. In that case, acute coronary syndrome with ST segment elevation developed following the use of a cold medicine and hypokinesis of the apical septum was detected on the magnetic resonance imaging of the left ventricle [6]. To our knowledge, our patient was the first case in whom recurrent Kounis syndrome developed due to pseudoephedrine use. Vasoactive mediators (histamine, leukotriene, and serotonin) and proteases (tryptase, chymase) are released both locally and into the systemic circulation by mast cell degranulation after contact with the allergens that have a role in the etiology [7]. Among these mediators, histamine and leukotrienes may lead to coronary events via vasoconstriction, whereas proteases lead to coronary events via metalloprotein activation, collagen reduction, and the potential of inducing atheromatous plaque erosion. Moreover, histamine plays a role in thrombocyte aggregation via thrombocyte activation [8, 9]. In our case, pseudoephedrine, a nonselective alpha- and beta-adrenergic receptor activator, might lead to myocardial injury by causing coronary artery spasm.

Kounis syndrome has two subtypes. Type 1 has no underlying coronary artery disease or risk factor. Vasoactive mediators that appear due to acute allergic reaction lead to coronary spasm. Chest pain secondary to ischemia and changes in ECG occur. Clinical picture may present in a wide spectrum ranging from unstable angina pectoris to sudden cardiac death. Cardiac enzymes may either be normal or reflect acute myocardial infarction. Type 2 comprises preexisting atheromatous plaque or coronary artery disease. Mediator that is released during acute allergic reaction may cause erosion or rupture of atheromatous plaque. In recent years, a third type has been defined due to widespread use of drug-eluting stents in percutaneous treatment of coronary artery disease. Histopathological examinations of thrombus material in patients presented with acute stent thrombosis have exposed the presence of eosinophils and mast cells [10]. The present case is consistent with type 1 Kounis syndrome since he had no known coronary artery disease or documented angiographic coronary artery stenosis or atheromatous plaque.

There is no specific pathognomonic test for the diagnosis of Kounis syndrome. The diagnosis is established based on symptoms and signs, ECG changes, laboratory findings, echocardiography, and angiography. Symptoms and signs of acute coronary syndrome and acute allergic reaction should suggest the diagnosis of Kounis syndrome. Patient with coronary syndrome may have angina-like chest pain. On the other hand, symptoms of allergic reaction may be encountered. Symptoms and signs of allergic reaction may include hypotension, pruritus, urticaria, dyspnea, wheezing, nausea, vomiting, and, in severe cases, angioedema after contacting with allergen.

Electrocardiographic findings in Kounis syndrome may be either in the form of nonspecific ST segment and T-wave alterations or normal or in the form of extensive ST segment depression and ST elevation. ST segment alteration is related to coronary vasospasm and most commonly involved derivations are those that reflect the inferior region. Angiographically, right coronary artery is involved most frequently.

Laboratory findings that indicate cardiac injury and allergic reaction are the other important analyses used in the diagnosis of Kounis syndrome. Troponin I/T, CK, CK-MB, and d-dimer are used for differential diagnosis among other causes of cardiac injury and chest pain. Analysis of tryptase concentration, histamine, arachidonic acid derivatives, interleukins, tumor necrosis factor, complement, eosinophil, and total and specific immunoglobulin E levels is recommended to specify the status of allergic reaction [11]. In the present case, the lack of analysis of immunological laboratory markers after pseudoephedrine use is the most significant limitation.

Echocardiography is the other diagnostic tool used in the differential diagnosis of chest pain in a patient with Kounis syndrome. Echocardiography may allow evaluation of left ventricular functions, aortic dissection, pericardial effusion, and acute pulmonary embolus. Regional contraction defects, which are usually reversible, may be encountered in patients with Kounis syndrome.

In Kounis syndrome, coronary angiography is performed both for diagnostic purpose and for percutaneous treatment, if necessary. In addition, subtype classification could be performed based on coronary angiographic findings.

Treatment of Kounis syndrome focuses on accompanying pathology and has no specific algorithm for treatment. Management of treatment of acute coronary syndrome is also valid for Kounis syndrome. Antiaggregant and anticoagulant treatments should be commenced in all cases with Kounis syndrome accompanied by acute coronary syndrome. Percutaneous intervention should be considered in cases having an underlying coronary lesion responsible for acute coronary syndrome. Angiotensin-converting enzyme inhibitors/angiotensin receptor blockers or beta-blockers can be used for long-term taking left ventricular functions into consideration [12].

Treatment of allergic reaction can be performed in accordance with the recommendations of allergy, asthma, and immunology societies [13]. The main goal of treatment should focus on mast cells that play a role in the physiopathology of Kounis syndrome. Corticosteroids, H1 and H2 blockers, and adrenalin have been used for this purpose [14]. The present case was referred to the Immunology Polyclinic for the investigation of sensitivity to pseudoephedrine or other agents and the decision of long-term antiallergic therapy.

Recurrent Kounis syndrome due to same agent is a quite rare condition. There are two cases in the literature. In the first case, it was observed that the patient, in whom clopidogrel treatment was commenced due to the diagnosis of acute coronary syndrome, developed chest pain and ST-T changes after clopidogrel. Similar findings were observed after the second clopidogrel given a day later [15]. In the second case, who was planned to undergo noncardiac surgical procedure under general anesthesia, the surgery was cancelled due to hypotension and ECG alterations developed after remifentanil. Similar clinical picture was observed when the patient again received remifentanil 1 month later for general anesthesia for the same surgery [16]. In the present case, pseudoephedrine use for two times at 8-month interval due to upper respiratory tract infection is in question. Chest pain and ECG changes were observed after pseudoephedrine use. Coronary arteries were normal on coronary angiography. Whilst there was a significant stenosis in coronary arteries in the aforementioned first case, coronary arteries were found to be normal in the second case.

In conclusion, allergic angina (Kounis syndrome) should be kept in mind in patients who present with acute coronary syndrome in case angiographic findings are inconsistent with clinical manifestation. Attention should be paid that recurrent use of or exposure to the agent that causes Kounis syndrome may lead to recurrent clinical pictures.
